# Supplementary material for: Comparison of the GLIM, ESPEN and ICD-10 Criteria to Diagnose Malnutrition and Predict 30-Day Outcomes: An Observational Study in an Oncology Population
Source: Nutrients. 2021 Jul 28;13(8):2602. doi: 10.3390/nu13082602 (PMC8402162; doi:10.3390/nu13082602)
Supplement: Supplementary file 1 [file nutrients-13-02602-s001.zip › nutrients-1299723-supplementary.pdf]

Supplementary Table S1: Diagnostic measures by ICD-10, ESPEN and GLIM

GLIM [c]	ESPEN	ICD-10
PHENOTYPIC CRITERIA<br>Low BMI (kg/m^2)<br><20 kg/m^2 (<70 years)<br>*OR*<br><22 kg/m^2 (≥70 years)<br><br>Unintentional loss of weight<br>>5%, ≤3 months<br>*OR*<br>>10%, ≥4 months<br><br>Reduced muscle mass [d]<br>(≥ 4 muscle sites rated as mild/moderate/severe deficit)<br><br>ETIOLOGICAL CRITERIA<br>Reduced food intake<br>≤50% usual intake for 5-30 days or ≥1month<br>or<br>≤75% usual intake for ≥1 month<br>or<br>>75% usual intake for ≥1 month<br><br>Inflammation/ disease burden<br>Presence of metastasis	OPTION ONE<br>Low BMI (kg/m^2)<br><18.5 kg/m^2<br><br>OPTION TWO<br>Unintentional loss of weight [b]<br>> 5% within 3 months<br>*OR*<br>>10% indefinite of time<br><br>Reduced BMI (kg/m^2)<br>< 20 kg/m^2 (<70 years)<br>*OR*<br>< 22 kg/m^2 (≥70 years)	OPTION ONE<br>Low BMI (kg/m^2)<br><18.5 kg/m^2<br><br>OPTION TWO [a]<br>Unintentional loss of weight<br>>5% indefinite of time<br><br>Reduced food intake<br>Any suboptimal intake of food<br><br>Muscle wasting<br>(≥ 4 muscle sites rated as mild/moderate/severe deficit)

Abbreviations: ICD-10, International classification of disease version 10 malnutrition criteria; ESPEN, European Society of Clinical Nutrition and Metabolism malnutrition criteria: GLIM, Global Leadership Initiative on Malnutrition criteria; BMI, body mass index. (a) Meet all of the following, (b) Combined unintentional weight and low BMI, (c) 1 phenotypic criterion and 1 etiological criterion needs to be present for diagnosis, (d) by a validated assessment method.

Supplementary Table S2: Additional details for each of the logistic regression models reported in Table 4

	Cox and Snell Nagelkerke R squared	Correctly classified/ total # of participants (%) of the model	Full model
**Mortality at 30-days**			
**GLIM**			
Patient type			
Metastasis			
Constant	0.056-0.237	2154/2221 (97.0%)	χ^2 (3, n=2221) = 128.60, p ≤0.001
**ESPEN**			
Patient type			
Metastasis			
Constant	0.060 – 0.234	2207/2285 (96.6%)	χ^2 (3, n=2284) = 141.79, p ≤0.001
**ICD-10**			
Patient type			
Metastasis			
Constant	0.048-0.209	2154/2219 (97.1%)	χ^2 (3, n = 2219) = 109.16, p ≤0.001
**Unplanned admission or readmission at 30-days**			
**GLIM**			
Patient type			
Metastasis			
Constant	0.029-0.052	1868/2168 (86.2%)	χ^2 (3, n=2168) = 63.22, p ≤0.001
**ICD-10**			
Patient type			
Metastasis			
Constant	0.026 – 0.047	1865/2164 (86.2%)	χ^2 (3, n=2164) = 57.43, p ≤0.001
**ESPEN**			
Patient type			
Metastasis			
Constant	0.023-0.041	1911/2225 (85.9%)	χ^2 (3, n=2225) = 51.93, p ≤0.001

Abbreviations: ICD-10, International classification of disease version 10 malnutrition criteria; ESPEN, European Society of Clinical Nutrition and Metabolism malnutrition criteria: GLIM, Global Leadership Initiative on Malnutrition criteria.
